# Type 2 Diabetes Complicated With Heart Failure: Research on Therapeutic Mechanism and Potential Drug Development Based on Insulin Signaling Pathway

**DOI:** 10.3389/fphar.2022.816588

**Published:** 2022-03-03

**Authors:** Hui Ye, Yanan He, Chuan Zheng, Fang Wang, Ming Yang, Junzhi Lin, Runchun Xu, Dingkun Zhang

**Affiliations:** ^1^ State Key Laboratory of Southwestern Chinese Medicine Resources, Pharmacy School, Chengdu University of Traditional Chinese Medicine, Chengdu, China; ^2^ TCM Regulating Metabolic Diseases Key Laboratory of Sichuan Province, Hospital of Chengdu University of Traditional Chinese Medicine, Chengdu, China; ^3^ State Key Laboratory of Innovation Medicine and High Efficiency and Energy Saving Pharmaceutical Equipment, Jiangxi University of Traditional Chinese Medicine, Nanchang, China

**Keywords:** type 2 diabetes, heart failure, insulin signaling pathway, myocardial energy metabolism, insulin resistance, drugs

## Abstract

Type 2 diabetes mellitus (T2DM) and heart failure (HF) are diseases characterized by high morbidity and mortality. They often occur simultaneously and increase the risk of each other. T2DM complicated with HF, as one of the most dangerous disease combinations in modern medicine, is more common in middle-aged and elderly people, making the treatment more difficult. At present, the combination of blood glucose control and anti-heart failure is a common therapy for patients with T2DM complicated with HF, but their effect is not ideal, and many hypoglycemic drugs have the risk of heart failure. Abnormal insulin signaling pathway, as a common pathogenic mechanism in T2DM and HF, could lead to pathological features such as insulin resistance (IR), myocardial energy metabolism disorders, and vascular endothelial disorders. The therapy based on the insulin signaling pathway may become a specific therapeutic target for T2DM patients with HF. Here, we reviewed the mechanisms and potential drugs of insulin signaling pathway in the treatment of T2DM complicated with HF, with a view to opening up a new perspective for the treatment of T2DM patients with HF and the research and development of new drugs.

**Graphical Abstract:**
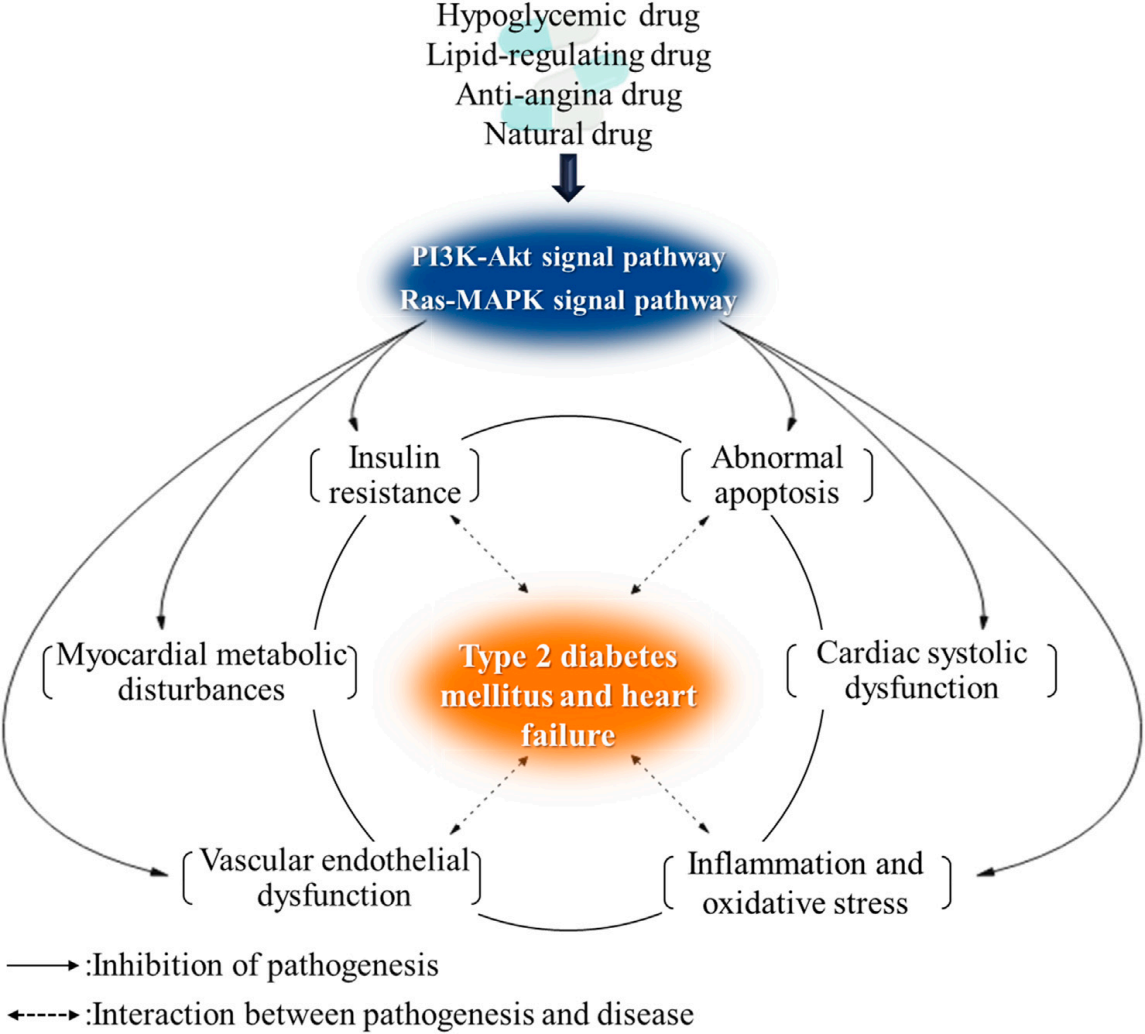
The insulin signaling pathway is a common signaling pathway to regulate myocardial energy metabolism and IR. The regulation of insulin signaling pathway will become an effective treatment for T2DM complicated with HF.

## Introduction

Type 2 diabetes mellitus (T2DM) and heart failure (HF) are common chronic diseases, which often occur simultaneously. About 415 million people worldwide suffer from diabetes, with T2DM accounting for more than 90% (Chatterjee et al., 2017). The annual meeting of the EASD (European Association for the Study of Diabetes) in 2004 pointed out that when T2DM occurs, patients have a first-degree HF. T2DM accelerates physiological cardiac aging through hyperglycemia and hyperinsulinemia (Sciacqua et al., 2021). Thus, T2DM complicated with HF is the final outcome in elderly patients with T2DM. The clinical incidence and mortality of T2DM complicated with HF are significantly higher than those of the general population, 4–8 times those of non-diabetic patients (Bell and David, 2004). Compared with patients without T2DM, patients with T2DM complicated with HF have more obvious clinical symptoms and signs, and their cardiac function classification, quality of life, and prognosis are worse (Seferović et al., 2018). Paolisso et al. (2021) also found patients with hyperglycemia *versus* normoglycemia had higher levels of inflammatory markers and B-type natriuretic peptide and lower left ventricular ejection fraction, and higher rates of heart failure and death. Acute hyperglycemia could induce a more severe depression of cardiac pump, with cardiac denervation, increased rate of acute HF, and worse prognosis at follow-up (Paolisso et al., 2021). Meanwhile, the intersection of T2DM and HF is mainly concentrated in elderly patients, which also increases the difficulty of its treatment.

Until recently, T2DM and HF have been managed independently. There is no effective therapy for T2DM complicated with HF, which is usually combined with blood glucose control and anti-HF therapy. The therapeutic drugs are very limited, and the effect is poor. Many hypoglycemic drugs have been found to have potential cardiovascular risks, which are not conducive to HF. And conventional drugs for HF treatment are also not effective in the treatment of DM, and it is still unclear whether they will aggravate the condition of diabetes or benefit the prognosis of diabetes. Therefore, it is necessary to further explore the treatment plan of DM combined with HF. Recently, sodium-dependent glucose transporter 2 inhibitors (SGLT2i), an oral glucose-lowering agent, have hit two birds (T2DM and HF) with a single stone (Santos-Ferreira et al., 2020). SGLT2i significantly reduced the rates of HF hospitalization in multiple cardiovascular outcome trials (CVOTs) (Marfella et al., 2021). Based on this evidence, the recent trend of SGLT2i has shifted from the prevention of HF in T2DM patients to the treatment of HF regardless of the presence of T2DM (Marfella et al., 2021). This also brings hope for drug therapy of T2DM complicated with HF.

The main pathological mechanism of T2DM complicated with HF is energy metabolism disorders and severe insulin resistance (IR). Early intervention to correct the metabolic disorder is an effective way to prevent the deterioration of the disease (Xiaoli and Shu, 2010). The insulin signaling pathway is a common signaling pathway to regulate myocardial energy metabolism and IR. Regulating this signaling pathway can improve a series of physiological and pathological mechanisms such as abnormal myocardial energy metabolism of HF, IR and vascular endothelial dysfunction, inflammation, and oxidative stress. Furthermore, it has the effect of reducing blood sugar and improving heart function, which is the best way to treat T2DM complicated with HF. In this paper, we reviewed the mechanisms and potential drugs of insulin signaling pathway in the treatment of T2DM complicated with HF to provide a comprehensive and detailed reference for relevant studies, and to contribute to the further research and development of new drugs for T2DM patients with HF.

## Insulin Signaling Pathway

There are two main pathways of insulin receptor signal transduction: 1) phosphatidylinositol 3-kinase/protein kinase B (PI3K/Akt) pathway, which is involved in the survival, growth, and proliferation of cells and protein synthesis and 2) RAS/mitogen-activated protein kinase (RAS-MAPK) pathway, which mainly regulates cell differentiation.

### Normal Insulin Signaling Pathway

**FIGURE 1:**
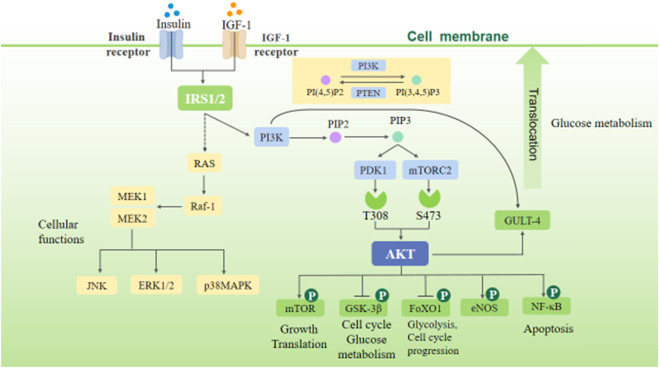
Insulin signaling pathway.

The insulin signaling pathway includes PI3K/Akt and RAS-MAPK signaling pathways. The binding of insulin or insulin-like growth factor (IGF-1) to the corresponding receptor can phosphorylate insulin substrate-1/2 (IRS-1/2), thus activating PI3K/Akt and RAS-MAPK signaling pathways (Figure 1). The PI3K/Akt signaling pathway is the most classical insulin signaling pathway, in which AKT phosphorylation is the key role. The phosphorylation of Akt leads to the phosphorylation of many downstream targets involved in cell growth and metabolism, including glycogen synthase kinase-3β (GSK-3β), glucose transporter-4 (GLUT-4), forkhead transcription factor O1 (FoxO1), endothelial nitric oxide synthase (eNOS), nuclear factor-κB (NF-κB), P21, and mammalian target of rapamycin (mTOR), thereby mediating the cell growth and proliferation, cell cycle regulation, and glucose metabolism regulation induced by insulin and various growth factors (Plotnikov et al., 2011). The RAS-MAPK signaling pathway can be indirectly activated by phosphorylation of IRS-1/2, thereby activating extracellular signal–regulated kinases (ERK1/2), c-Jun N-terminal kinase (JNK), and p38MAPK. These signals can activate downstream related factors and regulate many physiological processes such as cell growth, proliferation, differentiation, apoptosis, and immune response (Plotnikov et al., 2011).

(After insulin and IGF-1 activate, IRS, PI3K, and RAS are activated, and phosphorylation of PI3K activates Akt. These two important insulin signaling pathways can mediate cell growth, proliferation, differentiation, apoptosis, and immune response and regulate glucose metabolism and the energy metabolism, structure, and function of the heart.)

### The Change of Insulin Signaling Pathway in T2DM Patients With HF

In T2DM patients with HF, the insulin signaling pathway is obviously abnormal and accompanied by IR. In the early stage of T2DM with HF, the insulin signaling pathway is not significantly impaired. In the middle and late stages, the impairment of insulin signaling pathway is aggravated, accompanied by the synchronous aggravation of IR. In end-stage HF, the Akt signaling pathway is severely inhibited and the MAPK signaling pathway is partially active, accompanied by severe IR (Riehle and Abel, 2016).

Schulze et al. (Chokshi et al., 2012) found that myocardial Akt phosphorylation was significantly inhibited in the left ventricular samples from patients with advanced HF. After implanting the left ventricular assist device, the patient’s left ventricular function was significantly restored and the PI3K/Akt signaling cascade was activated. In addition, the activation of JNK and p38MAPK could induce apoptosis and necrosis, affect myocardial contractility, and deteriorate cardiac function (Bin, 2008). In both HF animal models and HF patients, the activities of JNK and p38MAPK in the failed myocardium are significantly higher than those in the healthy heart; also, the activities of JNK and p38MAPK in severe HF patients are also significantly higher than those in mild HF patients (Cook et al., 1999; Arabacilar and Marber, 2015). Significant improvement in cardiac function was observed after using p38MAPK inhibitors (Riba et al., 2017). These studies showed that the expression of insulin signaling pathway is abnormal during HF, especially in the end-stage HF. Meanwhile, the recovery of ventricular function is related to the activation of Akt signaling pathway and the inhibition of JNK and p38MAPK signaling pathways.

## Mechanism of Treating T2DM Complicated With HF Based on Insulin Signaling Pathway

T2DM complicated with HF has a complicated pathogenesis and involves multiple pathological mechanisms, including IR, myocardial energy metabolism disorders, vascular endothelial dysfunction, abnormal apoptosis of myocardial cells, abnormal calcium circulation of the myocardium, excessive inflammation of the myocardium, and oxidative stress. Regulating the insulin signaling pathway can activate/inhibit multiple targets, mediate the above-mentioned multiple mechanisms such as glucose and lipid metabolism, apoptosis, and inflammatory factor secretion, and improve T2DM complicated with HF.

### Insulin Signaling Pathway and IR

**FIGURE 2:**
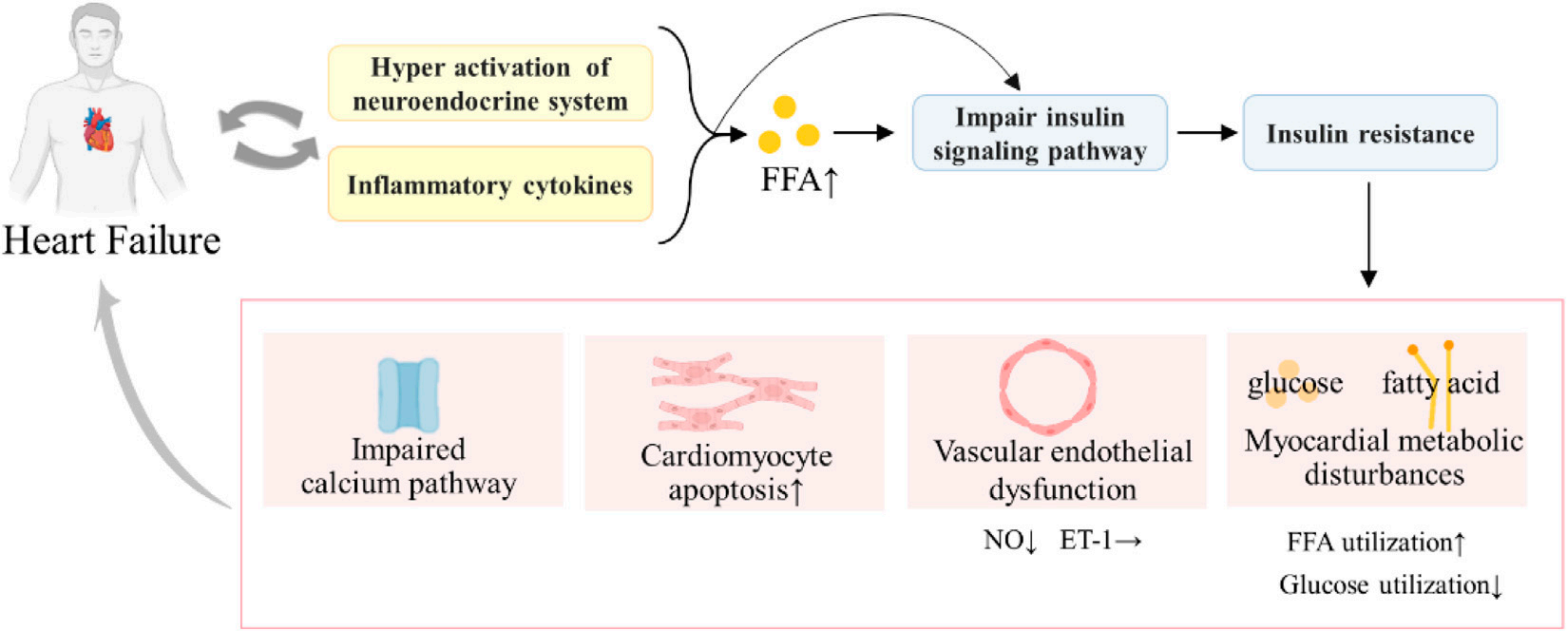
Relationship between insulin resistance and heart failure.

IR refers to a state in which insulin secretion is normal, but the sensitivity of tissues, organs, and cells to insulin is reduced and the role of insulin in promoting glucose uptake and utilization in target organs is reduced (Semenkovich, 2016). IR is a pathological feature of T2DM, as well as the main metabolic feature of pathophysiology of HF. Swan et al. (1997) found that the severity of chronic HF is significantly associated with increased IR, and patients with T2DM complicated with HF may have both systemic and cardiac IR. Furthermore, IR and T2DM complicated with HF are mutually causal, forming a vicious circle and worsening the disease (Figure 2).

The combined effect of systemic IR and myocardial IR can lead to a series of pathological injuries, such as impaired calcium signaling (Roe et al., 2013), substrate metabolism disorders, mitochondrial dysfunction (Qi et al., 2013) and oxidative stress, ER stress, and impaired cardiomyocyte endothelial nitric oxide (NO) signaling (Falskov et al., 2011). These pathological injuries could damage the heart, resulting in impaired calcium processing and contractility of the myocardium, decreased cardiac energy efficiency, myocardial cell apoptosis, and cardiac fibrosis (Aroor et al., 2012), accelerating the deterioration of T2DM complicated with HF (Nagoshi et al., 2011). Meanwhile, they can also aggravate IR, triggering a vicious cycle. With the development of T2DM complicated with HF, IR also aggravated. Especially in end-stage HF, patients are accompanied by severe IR. Therefore, improving IR can be a key entry point for treating T2DM complicated with HF.

Studies have shown that mitochondria play a major role in the pathogenesis of IR. An increase in mitochondrial oxidants is a consistent feature of IR *in vitro* and *in vivo* and in humans and can rapidly impair insulin-regulated GLUT4 translocation and significantly contribute to IR (Fazakerley et al., 2018). The mitochondrial oxidative stress and mitochondrial dysfunction are all related to IR (Fazakerley et al., 2018). Furthermore, the abnormality of insulin signaling pathway plays a key role in the occurrence and development of IR. IR can be induced by any abnormal insulin signal site, such as degradation, phosphorylation, and distribution of IRS, blocked activation and abnormal expression of PI3K, abnormal expression of PKD, Akt, GSK-3, and glucose transporters, reduction and activity disorder of glucose transporters, and activation of FoxO1 (Goedeke et al., 2019; Park et al., 2019). Therefore, it is beneficial for the early prevention and prognosis of T2DM complicated with HF to improve IR and break the vicious cycle of T2DM with HF by regulating the insulin signaling pathway.

(IR can induce HF through myocardial metabolic disorders, endothelial dysfunction, impaired calcium signaling transduction pathway, hormone endocrine abnormalities, inflammation, and oxidative stress. HF can also cause hyper-activation of the neuroendocrine system and inflammatory cytokine increases, which increase the free fatty acid (FFA) level and damage the insulin signaling pathway, thus leading to IR. HF and IR will form a vicious cycle, aggravating the disease.)

### Insulin Signaling Pathway and Energy Metabolism Disorders

There are severe systemic and myocardial glucose and lipid metabolism disorders in T2DM patients complicated with HF, resulting in significant abnormalities of myocardial energy metabolism and dysfunction of myocardial contraction. HF is a progressive disease. The metabolic substrates show different metabolic states in different stages and degrees of HF. In the early stage of HF, cardiomyocytes are in the compensatory stage, where the oxidative metabolic rate of FA remains normal or slightly increases, and glucose oxidation may increase to supplement myocardial energy deficiency (Aroor et al., 2012). In the early and middle stages of HF, there is no significant change in the utilization of myocardial energy substrates, and myocardial mitochondria may increase for compensation to maintain the overall quantity balance (Wang et al., 2009). In the late stage of HF, the heart loses its metabolic flexibility, where FA oxidation metabolism is significantly down-regulated, and the heart relies to a greater extent on glucose oxidation as a priority metabolic substrate (Aroor et al., 2012). Severe myocardial blood oxygen deficiency leads to IR. And glucose aerobic oxidation is reduced, and the energy supply mode is changed to glycolysis (Aroor et al., 2012). The ventricular remodeling process progresses to the decompensated state, myocardial energy is in a state of lack, and pyruvate produced by glycolysis is converted to lactic acid under hypoxia, further aggravating HF (Aroor et al., 2012). In addition, cardiomyocyte apoptosis and autophagy aggravate in the late stage of HF, and the cells cannot continue to maintain the compensatory effect of synthesizing mitochondria, which significantly reduces mitochondria and exacerbates energy metabolism disorders (Wang et al., 2009).

The insulin signaling pathway can improve abnormal energy metabolism in the whole body and myocardium by regulating the conversion of metabolic substrates and mitochondrial function. After activation of Akt, GLUT4 can be directly transferred from the cytosol to the plasma membrane to enhance glucose uptake of myocardial cells, as well as regulating the expression of genes such as GSK-3 and FoxO1 to promote myocardial glucose uptake and inhibit glycogen synthesis (Melenovsky et al., 2011; Jiménez-Osorio et al., 2016). In addition, stimulation of glucose oxidation not only increases energy supply in ischemic heart disease and HF but also increases heart function due to better coupling of glycolysis and glucose oxidation (Lopaschuk et al., 2010). Meanwhile, regulating the expression of GSK-3β, mTOR, and NF-κB can improve mitochondrial function and regulate myocardial mitochondrial metabolism. Parra et al. (2014) have confirmed that the activation of Akt–mTOR–NF-κB signaling pathway increased the Opa-1 protein level in myocardial cells both *in vitro* and *in vivo*, promoted mitochondrial fusion, increased the mitochondrial membrane potential, and increased the intracellular ATP level and oxygen consumption, thereby acutely regulating mitochondrial metabolism in myocardial cells.

Therefore, when cardiac energy metabolism has not been unbalanced in the early and middle stages of HF, the regulation of insulin signaling pathway could increase the aerobic oxidation of glucose and inhibit the acceleration of cardiomyocyte apoptosis to prevent mitochondrial dysfunction, thus effectively preventing the deterioration of HF. In the late stage of HF, the body has severe energy metabolism disorder accompanied by IR. The regulation of insulin signaling pathway could increase the aerobic oxidation of glucose, inhibit the aerobic oxidation of fatty acids, improve insulin sensitivity, improve IR, and improve mitochondrial function, which are the treatments for energy metabolism in the late stage of HF.

### Insulin Signaling Pathway and Vascular Endothelial Dysfunction

Vascular endothelial dysfunction (VED), a common pathological mechanism of diabetes and cardiovascular disease, directly affects the prognosis and survival of patients in the course of diabetes and cardiovascular disease. VED occurs early in the chain of atherosclerotic process and is a main determinant of acute coronary events, infarction, and reduction of cardiac pump (Harjola et al., 2020). VED can increase the whole systemic peripheral resistance and afterload through peripheral effects and aggravate the cardiac function impairment through central effects such as myocardial ischemia and inducible nitric oxide synthase (iNOS)-induced damaging effects (Penna et al., 2006). Nemoto et al. (2019) found that impaired endothelial function results in severe coronary artery stenosis as well as plaque vulnerability, while chronic hyperglycemia aggravates VED, resulting in more diffuse coronary artery lesions and worse clinical outcomes (Kang et al., 2018). Meanwhile, coronary heart disease and infarction are precipitants of acute HF, and VED under hyperglycemia is highly likely to cause acute HF and seriously increases cardiac mortality (Driscoll et al., 2017). Studies have also confirmed a higher rate of coronary endothelial dysfunction in DM patients, which is also the reason for the high incidence of HF and poor clinical outcomes in DM patients (Sardu et al., 2019a).

VED is mainly characterized by a reduction in the bioavailability of nitric oxide (NO) and excess production of reactive oxygen species (Quyyumi et al., 1995; Ren et al., 2017). Many diabetes and cardiovascular risk factors contribute to VED, such that hyperglycemia and IR in pre-DM might lead to endothelial dysfunction and worse prognosis by alterations in vasomotor tone and by the overproduction of inflammatory molecules and reactive oxygen species. And various cytokines produced in inflammation inhibit NO production and endothelial vasodilation by down-regulating the PI3K signaling pathway. The stabilization of vascular endothelial cell function and myocardial vasomotor capacity could be maintained by activating the Akt–eNOS signal axis and RAS–MAPK (ERK1/2)–ET-1 signaling pathway to mediate the production of vasodilator NO and vasoconstrictor ET-1, as well as maintaining the balance of NO and ET-1 (Kang et al., 2017). Akt1 was confirmed to be the key kinase of eNOS in the endothelial-specific Akt1-deleted adult mouse model, and eNOS function impairment reduced NO secretion and affected endothelial relaxation (Lee et al., 2018). In addition, it can regulate the insulin signaling pathway (GSK-3β, NF-κB, p38MAPK) to improve blood glucose homeostasis, IR, inflammation, and oxidative stress, thereby alleviating VED.

### Insulin Signaling Pathway and Apoptosis

T2DM is characterized by pancreatic *β* cell dysfunction, including hypofunction and the decrease of β-cells (Zhang et al., 2011). Studies have shown that β-cell replication can compensate for peripheral IR in patients with T2DM. However, the decreased expression of insulin receptor and the dysfunction of insulin signal transduction accelerated *β* apoptosis, which leads to the failure of β-cell proliferation and cannot compensate for peripheral IR to aggravate T2DM (Folli et al., 2011). Additionally, cardiomyocyte apoptosis and autophagy also occur at the end stage of HF, resulting in the reduction of the number of mitochondria and cardiomyocytes, which further accelerates the development of HF (Xiaomei and Guoliang, 2014).

The PI3K/Akt signaling pathway and MAPK signaling pathway can regulate the apoptosis of β-cells and cardiomyocytes, which is beneficial to improve islet function and slow the development of HF. The PI3K/AKT signaling pathway could affect the activation of a variety of downstream effector molecules, such as Bcl-2/Bcl-XL–associated death promoter (Shultz et al., 2010), FoxO1 (Gardino and Yaffe, 2011; Battiprolu et al., 2012; Qi et al., 2013; Guo, 2014), Caspase-9 (Shultz et al., 2010), and nuclear factor-κB (NF-κB), to produce anti-apoptotic effect in cells and promote cell survival. Both *in vitro* and *in vivo* experiments have shown that the activation of Akt signaling pathway can slow the pancreatic islet apoptosis of rats, prolong the survival of pancreatic islets, and remarkably improve its function of synthesizing and secreting insulin (Richards et al., 2005; Wang et al., 2011). In addition, Shiojima and Walsh (2002) showed that the activation of Akt can inhibit apoptosis of endothelial cells and promote neovascularization. The activation of JNK and p38MAPK signaling pathways promotes apoptosis, while the activation of ERK signaling pathway inhibits apoptosis.

### Insulin Signaling Pathway and Myocardial Contraction

A character of HF is increased leakage of Ca^2+^ from the sarcoplasmic reticulum (SR) *via* type 2 ryanodine receptor (RyR2) (Cho et al., 2016). The leaked Ca^2+^ in SR will be redistributed into the cytosol, thus increasing the cytoplasmic Ca^2+^ concentration, decreasing the electrochemical gradient inside and outside the cell, and impairing myocardial contractility (Cho et al., 2016). SR Ca^2+^ ATPase 2a (SERCA2a) is an important ATPase that can pump Ca^2+^ back into the SR of myocardial cells to mediate myocardial relaxation (Gorski et al., 2015). The decrease of myocardial SERCA level will severely impair systolic and diastolic functions. The activation of insulin signaling pathway can indirectly up-regulate the cellular calcium signaling pathway and regulate the intracellular Ca^2+^ concentration, thereby improving the myocardial contraction (Kim et al., 2008). Li et al. (2012) found that the mice with conditional cardiomyocyte-specific SERCA2 knockout would develop the end-stage HF with elevated [Na (+)] (i) and intracellular acidosis during 4 or 7 weeks. Studies have found that the activation of PI3K/Akt signaling pathway can enhance the function of L-type Ca^2+^ channels, up-regulate the activity of SERCA2a, regulate the calcium signaling pathway, increase the Ca^2+^ concentration, and maintain the normal heart beating (Aroor et al., 2012). A clinical trial (Jessup et al., 2011) also confirmed that the up-regulation of SERCA2a expression can improve myocardial contractility and is beneficial to HF treatment.

### Insulin Signaling Pathways and Inflammatory Reaction

Both T2DM and HF are regarded as inflammatory diseases. In patients with T2DM complicated with HF, the inflammatory markers such as TNF-α, IL-6, and CRP are significantly higher than those in healthy people (Pickup et al., 2000; Leinonen et al., 2003). In HFrEF and HFpEF patients, elevated circulating levels of pro-inflammatory cytokines and cytokine receptors were associated with mortality (Kenny and Abel, 2019). Therefore, early anti-inflammatory therapy can effectively prevent T2DM complicated with HF. PI3K/Akt/NF-κB, p38MAPK, and other insulin signaling pathways can regulate the production of inflammatory factors and ROS, play anti-inflammatory and antioxidant roles, and improve myocardial inflammation. Related studies (Maier et al., 2012) have shown that the NF-κB expression in patients with HF is significantly higher than that in healthy people, and the activity of NF-κB increased with the aggravation of HF. Watanabe et al. (2013) found that the inhibitor of NF-κB kinase (IKK) significantly alleviated myocarditis in myocarditis rats, as well as alleviating heart injury.

It has a close relationship of pathological mechanisms between T2DM and HF and can interact among IR, energy metabolism disorders, inflammation, oxidative stress, and endothelial dysfunction. Taking the regulation of insulin signaling pathway as the starting point, it can regulate a variety of pathological mechanisms of T2DM and HF at the same time, so as to delay the development of T2DM complicated with HF and improve the condition.

## Potential Drugs for the Treatment of T2DM Complicated With HF Based on Insulin Signaling Pathway

At present, marketed hypoglycemic drugs, lipid-lowering drugs, and anti-anginal drugs have found some drugs could regulate the insulin signaling pathway. These drugs can improve glucose and lipid metabolism, myocardial energy metabolism, and heart function of patients through the insulin signaling pathway.

### Synthetic Drugs

#### Hypoglycemic Drugs

Biguanides (metformin), glucagon-like peptide-1 (GLP-1) receptor agonists, and dipeptidyl peptidase-IV (DPP-4) inhibitors in hypoglycemic drugs can treat T2DM complicated with HF through the insulin signaling pathway and have little cardiovascular safety risk. So, they can be considered potential therapeutic agents for T2DM complicated with HF in the future. Table 1 summarizes the clinical research of the effect of these hypoglycemic drugs on T2DM and HF.

**TABLE 1:** Clinical application of hypoglycemic drugs in patients with HF and T2DM.

Drug	Clinical application						References
	Clinic types of experiments	Patients	Number of examinees (*n*)	Drug usage and dosage	Usage time	Main results and conclusion	
Metformin	Prospective study	HF patients with DM (mean age is 71.7 ± 7.8 years)	*n* = 1,519; metformin therapy group (*n* = 592)	/	/	Metformin therapy is associated with reduced mortality of HF patients with new-onset DM, mainly due to decreased cardiovascular mortality, and with a lower hospitalization rate.	Romero et al. (2013)
	A randomized, double-blind, placebo-controlled, crossover study	Treatment naive diabetic patients with chronic HF	*n* = 45	p.o., 2 g/day	3 months	Compared to placebo, metformin significantly reduced HbA1c and improved insulin sensibility. It confirms that metformin improves blood glucose control in patients with T2DM and chronic HF.	Stolarikova et al. (2018)
	A multicenter prospective study	Propensity score–matched patients with stable angina	*n* = 258	/	6 months	The patients with pre-DM had a higher percentage of endothelial LAD dysfunction as compared to patients with pre-DM treated with metformin. At the 24th month of follow-up, in pre-DM metformin patients, MACE was lower than that of pre-DM patients.	Sardu et al. (2019)
	A retrospective observational study	Patients with left ventricular hypertrophy (LVH)	*n* = 212	/	/	There is significant reduction in the incidence of HF in the metformin group compared to the non-metformin group (risk reduction 54%). And the metformin group did not develop any symptoms of HF. Metformin may delay the progression of early stages of HF to the advanced stage.	David and Droogan (2013)
Sitagliptin	A population-based study	Patients with diabetes (age ≥45 years)	*n* = 8,288	/	/	There were 935 events of hospitalization for HF (HHF), in which the association between the number of HHF events and the adherence to sitagliptin was linear. The use of sitagliptin was associated with a higher risk of HHF, but no excessive risk for mortality was observed.	Wang et al. (2014)
	A population-based, retrospective cohort study	Patients with diabetes and incident HF	*n* = 7,620; sitagliptin therapy group (*n* = 887)	/	/	Sitagliptin use was not associated with an increased risk of all-cause hospitalizations or death but was associated with an increased risk of HF-related hospitalizations among patients with T2DM with pre-existing HF.	Weir et al. (2014)
	A randomized controlled clinical trial	Patients with T2DM and HF	*n* = 36; experimental group (*n* = 18), control group (*n* = 18)	p.o., 100 mg/day	24 weeks	The blood glucose indicators FPG, 2hPG, HbAlc, and BMI in the experimental group were significantly lower than those in the control group. LVEF was higher than that in the control group, and the cardiac function and blood glucose were both improved.	Jin et al. (2014)
Exenatide	A double-blind, randomized controlled clinical trial	Patients with T2DM with congestive HF (CHF)	*n* = 20	i.v.gtt., 0.12 pmol/kg/min	6 h	Exenatide has rapid hemodynamic effects in male patients with type 2 diabetic CHF. Infusion of exenatide to patients will increase the cardiac index (CIP) due to time.	Nathanson et al. (2012)
	A randomized controlled trial	Patients with T2DM with HF	*n* = 2,389; exenatide therapy group (*n* = 1,161), placebo group (*n* = 1,228)	2 mg, once a week	/	The reduction in all-cause death or HHF was seen with exenatide in patients. And HHF was reduced in the exenatide group *versus* placebo.	Fudim et al. (2019)
	A randomized, double-blind, placebo-controlled, crossover study	Patients with ST-segment elevation myocardial infarction	*n* = 334; exenatide therapy group (*n* = 175), placebo group (*n* = 159)	/	/	Admission for HF was lower in the exenatide group (11%) compared to the placebo group (20%). All-cause mortality occurred in 14% in the exenatide group *versus* 9% in the placebo group.	Kyhl et al. (2016)
Liraglutide	A single-center, open-label, randomized, parallel-group, pilot study	T2DM patients with history of post-ischemic chronic HF	*n* = 32; liraglutide therapy group (*n* = 10), sitagliptin therapy group (*n* = 10), insulin glargine therapy group (*n* = 12)	i.h., establish tolerance to 1.8 mg/day	52 weeks	Only in liraglutide-treated patients, left ventricular end-systolic volume index (LVESVI) reduced and cardiac output and cardiac index increased significantly.	Arturi et al. (2017)
	A randomized, double-blinded, placebo-controlled multicenter trial	Patients with reduced LVEF ≤45%	*n* = 241; liraglutide therapy group (*n* = 122), placebo group (*n* = 119)	i.h., establish tolerance to 1.8 mg/day	24 weeks	Liraglutide did not affect left ventricular systolic function compared with placebo in stable chronic HF patients with and without diabetes. And liraglutide was associated with an increase in heart rate and more serious cardiac adverse events.	Jorsal et al. (2017)
	A multicenter, double-blind, randomized, placebo-controlled clinical trial	Stable chronic HF patients with and without DM	*n* = 541	i.h., establish tolerance to 1.8 mg/day	/	In LIVE, liraglutide significantly decreased hemoglobin A1c and increased heart rate and serious cardiac adverse events.	Liang and Gu (2020)

“/” means unclear; “q.d.” means once a day; “b.i.d.” means twice a day; “t.i.d.” means three times a day; “p.o.” means oral administration; “i.m.” means intramuscular injection; “i.v.” means intravenous injection; “i.v.gtt.” means intravenous infusion; “i.h.” means hypodermic injection.

##### Biguanides

Biguanides are classic oral hypoglycemic drugs, mainly including phenformin and metformin, but phenformin has been discontinued due to its severe lactic acidosis. Studies have confirmed that metformin is the first choice for the treatment of T2DM complicated with HF. Metformin could improve left ventricular remodeling and function by activating the AMP-activated protein kinase (AMPK) to increase glucose uptake and fatty acid oxidation, enhance insulin sensitivity, and reduce IR, thereby preventing the progression of HF (Nesti and Natali, 2017). Meanwhile, metformin can inhibit the activation of NF-κB by activating AMPK, thereby reducing the expression and release of inflammatory factors and adhesion molecules and thus reducing the incidence of inflammatory cardiovascular diseases (Kinsara and Ismail, 2018).

At present, there has been sufficient clinical evidence to prove that metformin can significantly reduce the glycosylated hemoglobin (HbA1c) level, mortality, all-cause rehospitalization rate, and cardiovascular rehospitalization rate in patients with DM complicated with HF (Stolarikova et al., 2018; Romero et al., 2013). Furthermore, metformin could also significantly improve the cardiac function and prognosis of patients. Especially in patients with end-stage HF, the one-year survival rate of the metformin group was significantly improved compared with that of the non-metformin group (Table 1) (Miaomiao and Shuang, 2018). Metformin therapy may reduce the high risk of cardiovascular events and the exacerbation of diabetic cardiomyopathy in pre-DM patients by reducing coronary endothelial dysfunction (Sardu et al., 2019).

In conclusion, although metformin can be used safely and effectively in T2DM patients complicated with HF, it should be noted that currently metformin is forbidden to be used in patients with acute or unstable HF. Patients with impaired liver and kidney function should be cautious about taking long-term use of metformin, as metformin can significantly limit liver and kidney clearance and may cause lactate poisoning (2018). But metformin can also be used in patients with stable congestive HF if patients’ renal function is normal (2018b). Patients with gastrointestinal reactions and other adverse reactions should be prevented and treated on time, as well as paying attention to regular examination of patients’ liver and kidney function.

##### Glucagon-Like Peptide-1 Receptor Agonists

In recent years, GLP-1 receptor agonists (GLP-1 RAs) have become the most attractive drug for diabetes. At present, exenatide and liraglutide have discovered the potential to treat T2DM complicated with HF. Other GLP-1 RAs have little cardiovascular research. Exenatide could up-regulate the expression of GLUT-4 and increase phosphorylation of AMPK and Akt to increase the glucose uptake of cardiomyocytes, thereby improving cardiac systolic function (Vyas et al., 2011). Exenatide can also enhance SR reuptake of Ca^2+^ by enhancing SERCA2a expression to improve calcium circulation (Harikumar et al., 2012). Liraglutide can mediate metabolism, vascular endothelial disorders, inflammation, endoplasmic reticulum stress, and other multiple pathways by inhibiting the activity of PPARγ, increasing the expression of AMPK (Koshibu et al., 2019), and inhibiting the JNK signaling pathway and caspase-12–mediated apoptosis signaling pathway (Li et al., 2019). In addition, serum-reduced GLP-1 levels were positively associated with IR (Ahmed et al., 2017). Studies have also confirmed that GLP-1 RAs (liraglutide) can reverse the increased phosphorylation of IRS1 serines, IKKα/β, and JNK in the model of IR and thus reverse the inhibited downstream mediators of IRS1 such as Akt, AS160, and GLUT4 translocation (Li et al., 2018). And liraglutide can also reduce the NF-KB activation, thus reducing inflammatory cytokines (Li et al., 2018). Importantly, GLP-1 may improve insulin sensitivity, glycemic control, and endothelial function, increase muscle microvascular perfusion, and stimulate angiogenesis, and these effects are retained in IR (Hagve et al., 2019; Love et al., 2020).

At present, GLP-1 agonists have been used for the treatment of chronic IR. They have beneficial cardiovascular effects for T2DM patients and are very suitable for T2DM patients with cardiomyopathy (Hagve et al., 2019; Love et al., 2020). Intervention of GLP-1 RA to CRTd therapy in diabetic patients significantly improved LVEF and the 6 min walking test, reduced the arrhythmic burden, and reduced hospital admissions for heart failure worsening by increasing the CRTd responder rate (Sardu et al., 2018). Clinical studies of exenatide and liraglutide show that GLP-1 RAs can reduce blood glucose as much as traditional hypoglycemic agents, and both can reduce hemoglobin A1c (Monami et al., 2014). Moreover, the incidence of hypoglycemia is much lower than that of sulfonylurea, insulin, and other drugs (Monami et al., 2014). However, it is unstable for these two drugs to improve left ventricular ejection fraction (LVEF) and other systolic function indicators in the clinical treatment of HF. In some clinic studies, it was found that exenatide and liraglutide not only failed to improve HF but even increased the heart rate (HR) and cardiovascular adverse events of patients (Table 1). Therefore, the cardiovascular safety of GLP-1 RA needs to be verified by more clinical studies.

#### Lipid-Lowering Drugs

Hyperlipidemia is closely related to atherosclerosis and is a risk factor for most cardiovascular diseases. Clinical studies have found that some lipid-lowering drugs can effectively treat HF and also have a certain effect on T2DM. Therefore, we discuss the commonly used lipid-lowering drugs based on the insulin signaling pathway—statins and fibrates.

##### Statins

Statins are the most important and widely used lipid-lowering drugs in the clinic. They are also widely used in atherosclerosis and are the most effective drugs for the prophylaxis and treatment of coronary heart disease. Most statins promote the production of NO in the myocardial vascular endothelium and improve vascular endothelial dysfunction in the IR state by up-regulating the expressions of Akt and P-Akt and PI3K/Akt/e NOS signaling pathway, thereby exerting vascular protection (Jie et al., 2014). Atorvastatin also can increase the activation of AMPK and inhibit FoxO1 to achieve the anti-myocardial hypertrophy effect (Park et al., 2018; Yu et al., 2018).

Statins have a remarkable clinical effect on HF (Table 2), which could significantly improve cardiac indicators (such as LVEF and HR), reduce the levels of myocardial inflammatory factors and risk of HF, and increase the risk of T2DM (Cederberg et al., 2015; Elmadbouh et al., 2015). Clinical trials have also found that statins cause the reduction of insulin sensitivity and IR and are associated with an increased risk of diabetes. Compared with placebo, statin therapy was associated with a 9% increased risk for incident diabetes (Sattar et al., 2010). Henna Cederberg (Cederberg et al., 2015) and Yaluri et al. (2016) found that both simvastatin and rosuvastatin can lead to insulin sensitivity and IR. Pravastatin, rosuvastatin, and other statins were also found to be related to the increase of DM (Culver et al., 2012; Ridker et al., 2012). Some studies also found that cardiovascular benefits of statins are far greater than those of DM. For instance, statins significantly reduced the primary major adverse cardiac endpoint in both high-risk DM participants and low-risk DM participants (Yandrapalli et al., 2019). The reason for this effect may be the difference in the dosage of statins. Considering the therapeutic effects of statins on HF and the potential risk of diabetes, it is clear that statins have a good prospect in the treatment of simple HF, which is worthy of further research. However, they are not suitable for the treatment of T2DM complicated with HF.

**TABLE 2:** Clinical application of lipid-lowering drugs in patients with HF and T2DM.

Drug	Clinical application						References
	Clinic types of experiments	Patients	Number of examinees (*n*)	Drug usage and dosage	Usage time	Main results and conclusion	
Atorvastatin	A randomized controlled clinical trial	Patients with asymptomatic HF after myocardial infarction	*n* = 162; observation group (*n* = 81), control group (*n* = 81)	p.o., 20 mg per night	12 months	TNFα, hs-CRP, IL-6, and other factors were improved in both groups, but the observation group showed greater results than the control group. Atorvastatin exerted a great effect in treating asymptomatic HF after myocardial infarction, which can evidently improve cardiac function and vascular endothelial function.	Wang et al. (2020)
	A follow-up study	Patients hospitalized for ischemic HF	*n* = 155; atorvastatin therapy group (*n* = 92)	/	/	The most frequent rehospitalization was in patients without statin therapy (66.7%), followed by patients on rosuvastatin (64.1%) and atorvastatin (13.2%). It confirms that statin therapy is associated with substantially better long-term outcomes in patients with HF.	Faris et al. (2018)
	A randomized controlled clinical trial	Non-ischemic chronic HF patients	*n* = 40; control group (*n* = 20), experimental group (*n* = 20)	p.o., 40 mg/day	6 weeks	In patients, atorvastatin improved heart function E/A velocity ratio; decreased LV-end diastolic diameter (LV-EDD) and LV-end systolic diameter (LV-ESD), and significantly reduced serum lipid profiles, cTnT, hs-CRP, and MDA *versus* patient controls.	Elmadbouh et al. (2015)
Rosuvastatin	A randomized, double-blind, placebo-controlled trial	Patients with chronic HF	*n* = 4,574; rosuvastatin therapy group (*n* = 2,285), placebo group (*n* = 2,289)	p.o., 10 mg/day	Followed up for a median of 3.9 years	1,305 (57%) patients in the rosuvastatin group and 1,283 (56%) in the placebo group died or were admitted to the hospital for cardiovascular reasons. It confirms that rosuvastatin 10 mg daily did not affect clinical outcomes in patients with chronic HF of any cause.	Mitrohina and Kuryata (2008)
	/	Non-diabetic participants	*n* = 8,749; participants on statin treatment (*n* = 2,142)	/	/	Participants on statin treatment had a 46% increased risk of T2DM. Insulin sensitivity was decreased by 24% and insulin secretion by 12% in individuals on statin treatment compared with individuals without statin treatment.	Cederberg et al. (2015)
	/	Patients with systolic HF (age ≥60 years)	*n* = 5,011	/	/	Rosuvastatin was shown to reduce the risk of HHF by approximately 15%–20%, equating to approximately 76 fewer admissions per 1,000 patients treated over a median 33 months of follow-up.	Rogers et al. (2014)
Fluvastatin	/	Patients with ischemic HF and hyperlipidemia	*n* = 29	p.o., 80 mg/day	3 months	Compared with those of healthy subjects, the heart rate recovery (HRR) values were significantly lower in the HF patients in both the 1st and 3rd minutes. The results revealed an improvement in HRR in HF patients by fluvastatin treatment.	Katircibasi et al. (2005)
	A prospective uncontrolled study	Patients with ischemic HF	*n* = 29	p.o., 80 mg/day	12 weeks	After fluvastatin therapy, levels of IL-10 in the plasma were significantly increased and plasma TNF-α levels were significantly decreased. Fluvastatin therapy significantly improved HRR at 1 min after 12 weeks compared with baseline.	Tekin et al. (2016)
	An open label and prospective study	HF patients with idiopathic dilated cardiomyopathy (DCM) and ischemic cardiomyopathy (ICM)	*n* = 40; DCM group (*n* = 20), ICM group (*n* = 20)	p.o., 80 mg/day	12 weeks	After fluvastatin therapy, functional capacity and LVEF improved and the levels of TNF-α and IL-6 decreased. The results revealed fluvastatin improved cardiac functions and the clinical symptoms in HF patients with either idiopathic dilated or ischemic etiology.	Gürgün et al. (2008)
Simvastatin	A randomized, double-blind, placebo-controlled trial	Patients with coronary heart disease without evidence of HF	*n* = 4,444; placebo group (*n* = 2,223), simvastatin therapy group (*n* = 2,221)	p.o., 20 mg–40 mg	Followed for more than 5 years	Mortality was 31.9% in the placebo group and 25.5% in the simvastatin group among patients who developed HF. There were 45 hospitalizations because of acute HF in the placebo group and 23 in the simvastatin group. This indicates that long-term prevention with simvastatin reduces the occurrence of HF in a cohort of patients with coronary heart disease without previous evidence of CHF.	Kjekshus et al. (1997)
	An open non-randomized study	Patients with diastolic chronic HF	*n* = 125; main group (*n* = 66), control group (*n* = 59)	/	6 months	Significant increase in E (peak early diastolic left ventricular filling velocity) value by 14.1% and E/A (A peak left ventricular filling velocity at atrial contraction) ratio by 18.7% was found in the main group. It confirms that simvаstatin therapy resulted in significant improvement in the left ventricle diastolic function.	Pinchuk et al. (2015)
	A randomized, double-blind, controlled trial	Patients with chronic HF and preserved systolic function	*n* = 34; study group 1 (*n* = 20), control group 2 (*n* = 14)	p.o., 10 mg/day	12 weeks	After 12 weeks of treatment with simvastatin, insulin levels in 30% patients have decreased in group 1 by 26.47% and HOMA index by 28.78% and in 19% patients in group 2 by 9.47 and 9.76%, respectively. It confirms that simvastatin is effective and safe for patients with chronic HF and preserved systolic function and reduces IR.	Mitrohina and Kuryata (2008)
Fenofibrate	/	Patients with chronic HF	ICM patients (*n* = 57), DCM patients (*n* = 71)	/	/	In circulating angiogenic cells (CACs), fibronectin adhesion function was reversed by FF treatment, suggesting that FF reversed CACs and late EPC dysfunction in chronic HF patients.	Huang et al. (2020)
	A randomized, double-blind, controlled trial	Patients with chronic HF	*n* = 70; standard therapy group (*n* = 35), FF therapy group (*n* = 35)	p.o., 0.2 g q.d.	6 months	After FF combined with standard therapy, the patients’ serum PC Ⅰ, PC Ⅲ, LN, and HA concentrations decreased significantly, and the decrease was greater than that in the standard therapy alone. At the same time, the systolic and diastolic functions of the patient were significantly improved.	Xiaoyan et al. (2013)
	A randomized controlled clinical trial	Elderly patients with chronic HF(age 62–75 years)	*n* = 32; FF therapy group (*n* = 16), control group (*n* = 16)	200 mg every night	6 months	After 90 days of treatment, the NYHA classification, 6MWD, TG, and BUA levels of the FF group improved better than those in the control group. The LVEF and LVEDD of the FF group were significantly improved compared with those in the control group after 180 days of treatment.	Zhiming and Zhonghua (2011)

##### Fibrates

At present, fenofibrate (FF) is the only fibrate drug found to be able to treat HF by the insulin signaling pathway. It can enhance the oxidative metabolism of free fatty acids by up-regulating the expression of myocardial PPARα and FoxO1 (Ramakrishnan et al., 2016) to improve the energy metabolism of failed myocardium, inhibit ventricular remodeling (Li et al., 2015), and inhibit cardiomyocyte hypertrophy in multiple ways (Le et al., 2012). Meanwhile, FF down-regulates the activity of phosphokinase and phosphatase-1 in mitochondria, thus prolonging insulin action to improve its sensitivity and alleviate IR (Huang et al., 2017). Clinical studies have shown that FF has a good effect on HF patients (Table 2), which can significantly improve cardiac function indicators and myocardial systolic and diastolic functions. However, there is no clinical study of FF in the treatment of T2DM complicated with HF.

The bioavailability of FF is low, the patient’s compliance is poor, and there are large individual differences (Chang and Guangyu, 2021). In order to solve the problem of low bioavailability of FF, micro-powder technology, nanocrystallization technology, self-emulsifying technology, and slow and controlled release technology have been developed to improve the dissolution of FF dosage form. For example, the Fournier Laboratories developed the FF suprabioavailable tablet which can increase bioavailability by 25% *via* combining micronization technology with a new micro-coating process (Guichard et al., 2000). FF has shown good safety in long-term clinical application, with a low incidence of adverse reactions, most of which are mild. Considering its efficacy and safety, FF is expected to be a therapeutic agent for T2DM complicated with HF, but its clinical study in T2DM complicated with HF needs to be expanded.

##### Dipeptidyl Peptidase-IV Inhibitors

Dipeptidyl peptidase-IV (DPP-4) inhibitors are hypoglycemic drugs, commonly used in patients with T2DM. Some DPP-4 inhibitors have a protective effect on the heart. For example, sitagliptin can increase IRS-2 mRNA, inhibit PI3K-p85α protein expression, improve the IRS-2/PI3K signaling pathway, reduce IR, activate the AMPK signaling pathway, and inhibit the MAPK signaling pathway and NF-κB signaling pathway to reduce inflammatory response and cardiovascular diseases (Zheng et al., 2018). However, clinical studies (Table 1) show that DPP-4 inhibitors are generally not safe and have a potential cardiovascular risk profile. For instance, alogliptin has a higher risk of HF (Guo et al., 2017). Although sitagliptin and saxagliptin did not increase the risk of HF, they will increase the hospitalization rate of HF patients (Bowes et al., 2019). Furthermore, some studies illustrated that sitagliptin increases the risk of HF and the hospitalization rate of HF in DM patients (Weir et al., 2014). In 2013, the European Congress of Cardiology conducted a study, and the results showed that 20.1% (1,672) patients treated with saxagliptin and 19.6% (1,643) patients treated with placebo experienced cardiovascular adverse events. 3.5% (289) patients in the group treated with saxagliptin and 2.8% (228) patients in the group treated with placebo were hospitalized due to HF. The results suggested that saxagliptin did not increase the occurrence of ischemic cardiovascular events, but it inclined to increased hospitalization for HF in patients with the already present HF (Spinar and Smahelová, 2013). Based on the cardiovascular safety of DPP-4 inhibitors, sitagliptin, saxagliptin, and vildagliptin with low cardiovascular risk are recommended to be used as second-line or third-line additional treatment for T2DM combined with HF, which cannot increase the risk of HF, hypoglycemia, or death.

Diabetes is a common pathogenesis basis and risk factor of HF, and its complication rate is high clinically. Hypoglycemic drugs that treat HF through the insulin signaling pathway will be suitable for treating T2DM complicated with HF, which greatly reduces the incidence risk of HF and improves the therapeutic effect compared with simple HF treatment. Although hypoglycemic agents can regulate metabolism through the insulin signaling pathway, not all are suitable for the treatment of HF. Some hypoglycemic agents can cause adverse effects on the cardiovascular system, such as causing arrhythmia and HF. Furthermore, some drugs can treat HF, but there is a lack of clinical research on the treatment of T2DM combined with HF, and their safety needs to be investigated. Therefore, the hypoglycemic agents for HF need to be strictly screened.

#### Anti-Anginal—Trimetazidine

The therapeutic effect of anti-anginal drugs is worthy of affirmation for cardiovascular disease. Trimetazidine (TMZ), the first-line anti-angina drug, can treat HF through the insulin signaling pathway. Recent guidelines published by ESC 2016 have recommended TMZ in patients with HF with ongoing angina (Dalal and Mishra, 2017). TMZ can prevent reperfusion-mediated heart injury and dysfunction by activating insulin signaling pathways such as p38MAPK and Akt and regulating the expression of miRNA-21 in cardiomyocytes both *in vivo* and *in vitro*, thereby reducing cardiomyocyte apoptosis and enhancing anti-inflammatory and antioxidant capacity (Khan et al., 2010; Gao et al., 2011). Clinical studies of TMZ in HF treatment (Table 3) showed that it can indeed significantly improve the blood glucose and left ventricle function of patients and can significantly improve HbA1c, the blood glucose level, LVEF, the myocardial velocity of the left ventricle (LV) and right ventricle (RV), and other indicators. For T2DM patients with HF, the clinical effect of TMZ is more significant.

**TABLE 3:** Clinical application of anti-anginal drugs in patients with HF and T2DM.

Drug	Clinical application						References
	Clinic types of experiments	Patients	Number of examinees (*n*)	Drug usage and dosage	Usage time	Main results and conclusion	
Trimetazidine	A meta-analysis of randomized controlled trials	Patients with chronic HF	*n* = 994; TMZ therapy group (*n* = 80), placebo therapy group (*n* = 76)	/	/	Treatment with TMZ also resulted in significant decrease in LVESV, LVEDV, hospitalization for cardiac causes, and B-type natriuretic peptide. However, there were no significant differences in exercise duration and all-cause mortality between patients treated with TMZ and placebo.	Zhou et al. (2014)
	A prospective, single-blind and single-center study	HF patients	*n* = 87; TMZ therapy group (*n* = 51), placebo therapy group (*n* = 36)	p.o., 20 mg t.i.d.	3 months	Compared to placebo, increments in LVEF and myocardial velocities were significantly higher with TMZ. An increase in LVEF with TMZ was significantly correlated with the presence of DM. It is suggested that addition of trimetazidine to current treatment of HF, especially for those who are diabetic, may improve LV and RV functions.	Gunes et al. (2009)
	A prospective, observational, non-interventional, open-label clinical study	Patients with stable angina pectoris and T2DM	*n* = 737	p.o., 35 mg t.i.d. Patients with moderate renal impairment received TMZ 35 mg q.d.	6 months	TMZ treatment significantly improved glucose metabolism, lowered HbA1c and glucose levels, and decreased arterial stiffness. In most patients, the tolerability of trimetazidine was rated as excellent to good, with a low incidence of adverse events.	Meiszterics et al. (2017)

In clinics, TMZ ordinary tablets and sustained-release tablets were commonly used. Compared with ordinary tablets, sustained-release tablets can delay the release of TMZ, ensure a uniform and constant blood concentration, avoid peaking the problem of blood concentration, and make the anti-myocardial ischemia effect more durable, effectively covering the early morning dangerous period (Meirong et al., 2012). In addition, TMZ is well tolerated, with only a few cases of adverse reactions (such as nausea and vomiting). Considering the efficacy and safety, the clinical research of TMZ in HF is relatively mature, and it is recommended for the treatment of T2DM complicated with HF.

Among the above synthetic drugs, metformin and trimetazidine have good efficacy and high safety in treating T2DM complicated with HF, which can be extended to the clinical treatment of T2DM complicated with HF after improvement. Although GLP-1 agonists can be partially effective in T2DM complicated with HF, the efficacy is poor. Therefore, more effective derivatives or prodrugs need to be found. Metformin, trimetazidine, and fenofibrate have good efficacy in the treatment of HF, but there are defects such as low bioavailability and gastrointestinal adverse reactions. Therefore, they can be modified in the structure or dosage form to improve their bioavailability and reduce adverse reactions. For example, metformin has poor lipophilic property and low bioavailability and may cause gastrointestinal discomfort in some patients (Mingji, 2014). In order to improve the lipophilic property, bioavailability, and safety of metformin, it is necessary to modify the structure of metformin by introducing some lipophilic groups. Huttunen et al. (2013) synthesized several lipophilic sulfenamide prodrugs of metformin, and the oral absorption of these drugs was greatly improved compared with that of metformin. The bioavailability of prodrug 2a in rats is increased from 43 to 65%, and with increased oral absorption, the dose of metformin could be reduced, thereby reducing some of the adverse reactions (Huttunen et al., 2009). In addition, the absorbability of different dosage forms of FF *in vivo* is quite different. The bioavailability of common dosage form FF is 60%–90%, while the bioavailability of FF can be increased by 30% after micronization (Kai et al., 2010).

### Natural Products

Natural drugs and their active components have the advantages of multiple targets, comprehensive curative effect, small side effects, high safety, etc., which can make up for the defects of synthetic drugs such as single action and more adverse reactions. At least 16 natural products have been found to be capable of treating DM or HF *via* the insulin signaling pathway, such as astragaloside IV (Shi-Guang et al., 2015; Zhiwei et al., 2017), tanshinone IIA (Hwang et al., 2013; Yuan et al., 2014), and salvianolic acid A (Chen et al., 2016; Xue-Li et al., 2019), but only sodium ferulate (SF), tetramethylpyrazine (TMP), and resveratrol; TMP and resveratrol can be used for both DM and HF. These three natural products have been clinically used in T2DM and HF, respectively (Table 4), and they have great potential for the treatment of T2DM complicated with HF.

**TABLE 4:** Clinical application of natural drugs in patients with HF and T2DM.

Drug	Clinical application						References
	Clinic types of experiments	Patients	Number of examinees (n)	Drug usage and dosage	Usage time	Main results and conclusion	
SF	/	Patients with chronic pulmonary heart disease and HF	*n* = 31	Venous drop, SF and glucose injection 200 ml (200 mg), q.d.	10 days	In the SF treatment group, 28 cases were markedly effective, with a total effective rate of 90.3%. Most patients’ symptoms were improved, blood gas indexes were normal or improved, and no adverse reactions occurred.	Song (2007)
	A randomized controlled clinical trial	Patients with diabetic cardiomyopathy	*n* = 60	i.v.gtt., sodium ferulate injection (0.3 g), q.d.	30 days	In the SF group, EF and E peak/A peak were significantly higher after treatment than before treatment. SF combined with basic medication and insulin subcutaneous injection can effectively reduce blood lipids, improve heart function, and effectively alleviate the symptoms of DCM.	Fengxiang (2014)
TMP	/	DM patients	*n* = 16	i.v.gtt., TMP injection (LJ) 250 ml (5 mg/kg), q.d.	20 days	Whole blood viscosity at 3.75 s shear rate was decreased, ADP-induced PA was decreased, and ED was slightly changed. It indicated that TMP could be a potential medication to ameliorate or prevent chronic vascular complications in diabetes.	Zhao et al. (1989)
	/	Patients with chronic congestive HF	*n* = 84	i.v.gtt., *Astragalus* injection 250 ml q.d., LJ (400 mg) 250 ml q.d.	14 days	The combination of TMP injection and *Astragalus membranaceus* is more effective in the treatment of HF and can significantly improve the symptoms of HF.	Zhen and Faxiang (2011)
Resveratrol	/	HF patients	*n* = 59	p.o., resveratrol 4 mg every night	2 months	Serum total cholesterol (TC), low-density lipoprotein cholesterol (LDL-C), and CRP were all reduced to varying degrees, while LVEF was significantly increased, and the number of hospitalizations, total days, and mortality were reduced compared with those in the control group.	Guoping et al. (2005)
	A randomized, double-blind, placebo-controlled trial	Patients with T2DM and coronary heart disease (CHD)	*n* = 56; resveratrol therapy group (*n* = 28), placebo group (*n* = 28)	p.o., resveratrol 500 mg/day	4 weeks	Our-week supplementation of resveratrol in patients with T2DM and CHD had beneficial effects on glycemic control, HDL-cholesterol levels, the total/HDL-cholesterol ratio, and TAC and MDA levels.	Hoseini et al. (2019)

#### Sodium Ferulate

SF, with the chemical name 3-methoxy-4-hydroxycinnamate sodium, is the sodium salt of ferulate acid, which is the main component of *Angelica sinensis* (Oliv.) Diels and *Ligusticum chuanxiong* Hort. Studies have confirmed that SF can induce mkP-1 protein expression to alleviate myocardial hypertrophy caused by angina pectoris by inhibiting the expression of PKC-β, RAF-1, and p-ERK1/2 protein and regulating MAPK/ERK and JNK pathways (Luo et al., 2019; Hu et al., 2017). For the clinical treatment of cardiovascular diseases, SF sodium chloride injection and SF glucose injection are mainly used in the adjuvant treatment of vascular diseases such as atherosclerosis, coronary heart disease, and vasculitis. Clinical data also demonstrate that SF works well in the treatment of T2DM and HF (Table 4), and SF, with low toxicity and good safety, is easy to be metabolized by the human body. However, SF aqueous solution is unstable to light and easily decomposes, which may cause adverse reactions due to its impurities (Yanhua et al., 2011). Therefore, it is suggested to take measures to avoid light to increase drug stability and reduce adverse drug reactions.

#### Tetramethylpyrazine

TMP is an alkaloid extracted from *Ligusticum chuanxiong* Hort. Studies have shown that TMP mainly activates the Akt and AMPK signaling pathways, up-regulates the expressions of GLUT-4 and eNOS to mediate the recovery of insulin signal transduction, reduces IR, increases the utilization of glucose by cardiomyocytes, and regulates apoptosis and autophagy, thereby improving cardiac function (Yanhua et al., 2011; Lv et al., 2012; Rai et al., 2019). At present, TMP phosphate tablets, TMP phosphate capsules, TMP phosphate injection (only for intramuscular injection), TMP hydrochloride injection, and other dosage forms are mainly used clinically. The combination of TMP injection and *Astragalus membranaceus* (Fisch.) had a better effect on HF and can significantly improve the symptoms of HF (Zhen and Faxiang, 2011). *Salvia miltiorrhiza* and ligustrazine injection can also effectively improve the left ventricular function and myocardial systolic function in the treatment of chronic congestive HF with high safety (Wenhhhe, 2016).

There are many TMP derivatives, including TMP ether derivatives, ester derivatives, piperazine derivatives, and stilbene derivatives (such as resveratrol), most of which show a certain cardiovascular protection (Zou et al., 2018). For example, TMP-amide derivatives have protective effects on injured vascular endothelial cells; TMP-benzyl alcohol ether derivatives mostly protect against ischemia–reperfusion injury and improve vasodilation (Zhen and Faxiang, 2011; Wenhhhe, 2016). Many studies have modified the structure of TMP and found that the pyrazine ring (parent nucleus) of TMP may be its pharmacodynamic group, and the side chain is its pharmacokinetic group, which mainly affects the absorption, distribution, and metabolism of TMP *in vivo* (Zou et al., 2018). On the basis of retaining the parent nucleus, the side chain of TMP can be modified to obtain cardiovascular drugs with lower toxicity, better efficacy, better water solubility, and higher bioavailability (Chen et al., 2011). Chen et al. (2011) synthesized a series of novel ligustrazinyloxy-cinnamic acid derivatives, several of which showed enhanced inhibitory effect on platelet aggregation compared with TMP.

#### Resveratrol

Resveratrol, a representative drug of ligustrazine-stilbene derivatives, has pharmacological effects on regulating the blood lipid level, preventing oxidation of low-density lipoprotein and anti-platelet agglutination, and reducing the incidence of sudden heart disease (Li et al., 2016). Studies confirmed that resveratrol mainly activates p38MAPK-β, Akt, NF-κB, and PI3K/Akt/mTOR signaling pathways and inhibits PI3K/Akt/FoxO3a and p38MAPK-α signaling pathways to regulate apoptosis and related inflammatory factors, thereby exerting myocardial protection (Das et al., 2006; Wu et al., 2017; Xiuling et al., 2019). Although resveratrol is highly absorbed when given orally, it has a very low bioavailability due to rapid metabolism of its glucuronide and sulfate conjugates (Walle et al., 2004). Therefore, its effective dosage is not accurate. When the dosage is low (5 μmol/L–20 μmol/L), it can protect the myocardium from ROS damage. It will promote oxidation and cause adverse reactions at higher dosages, such as liver and kidney toxicity (Zordoky et al., 2015). A study has shown that resveratrol has been shown to inhibit angiogenesis at 30 mg/kg/day or higher doses (Zordoky et al., 2015).

In summary, SF, TMP, and resveratrol have low side effects, high safety, and mature clinical application, so we can consider increasing their clinical verification as potential therapeutic drugs for T2DM complicated with HF in the future. Many *in vitro* studies have shown that promising active components of natural drugs have been abandoned due to extremely low or even no activity or high toxicity *in vivo*. The reason for low activity *in vivo* may be due to its unsatisfactory pharmacokinetic properties, such as strong first-pass effect, low bioavailability, short half-life, rapid metabolism, and difficulty in entering target organs through biofilm (Shu, 2005). The bioavailability in the active ingredients of natural drugs is low, and the clinical dosage needs to be increased. It will inevitably increase the clinical risk and the number/amount of patients taking the drugs and is not conducive to clinical safety and patient compliance. The same is true of sodium ferulate, ligustrazine, and resveratrol. At present, these defects can be improved through structural modification and dosage form change. For example, Peng et al. (Xiaoyun et al., 2005) prepared ligustrazine phosphate dropping pills, which significantly improved its bioavailability and inevitably reduced its clinical risk. Peñalva et al. (2018) wrapped resveratrol with casein nanoparticles and found that its oral bioavailability is 26.5%, which was 10 times higher than when the resveratrol was administered as an oral solution. The modification of dosage form for natural drugs can improve its bioavailability, reduce the number and dose of drugs taken by patients every day, and improve patients’ compliance. Meanwhile, this way can also reduce clinical risk and improve efficacy, which is suitable for long-term medication of T2DM complicated with HF and other cardiovascular diseases.

### Other Drugs

Many new drugs currently in the research and development stage have the potential to treat cardiovascular diseases through the insulin signaling pathway. Poxel SA’s Imeglimin, a new T2DM drug, has successfully completed phase 3 development in Japan. Marianne et al. (Lachaux et al., 2020) found that Imeglimin immediately countered metabolic syndrome–related cardiac diastolic and vascular dysfunction in a rat model with human metabolic syndrome by reducing oxidative stress/increased NO bioavailability and improving myocardial perfusion. After 90-day treatment, myocardial and kidney structures in rats were improved after 90-day treatment (Lachaux et al., 2020). Esperion’s bempedoic acid, a new type of non-statin lipid-lowering drug, affects glucose and lipid metabolism and inhibits fatty acid and cholesterol synthesis by activating AMPK and has completed a phase 3 clinical trial. Masson et al. (2020) found that bempedoic acid significantly can reduce the levels of all atherogenic lipid markers, including LDL-C, non-HDL-C, and apolipoprotein B, and prevent atherosclerosis. Therefore, we can explore potential drugs to reduce cardiovascular risk in new drugs such as hypoglycemic drugs and lipid-lowering drugs, which also provides direction and convenience for the research and development of drugs based on the insulin signaling pathway to treat T2DM complicated with HF.

### Integration Analysis of Drug, Target, and Metabolic Pathway

**FIGURE 3:**
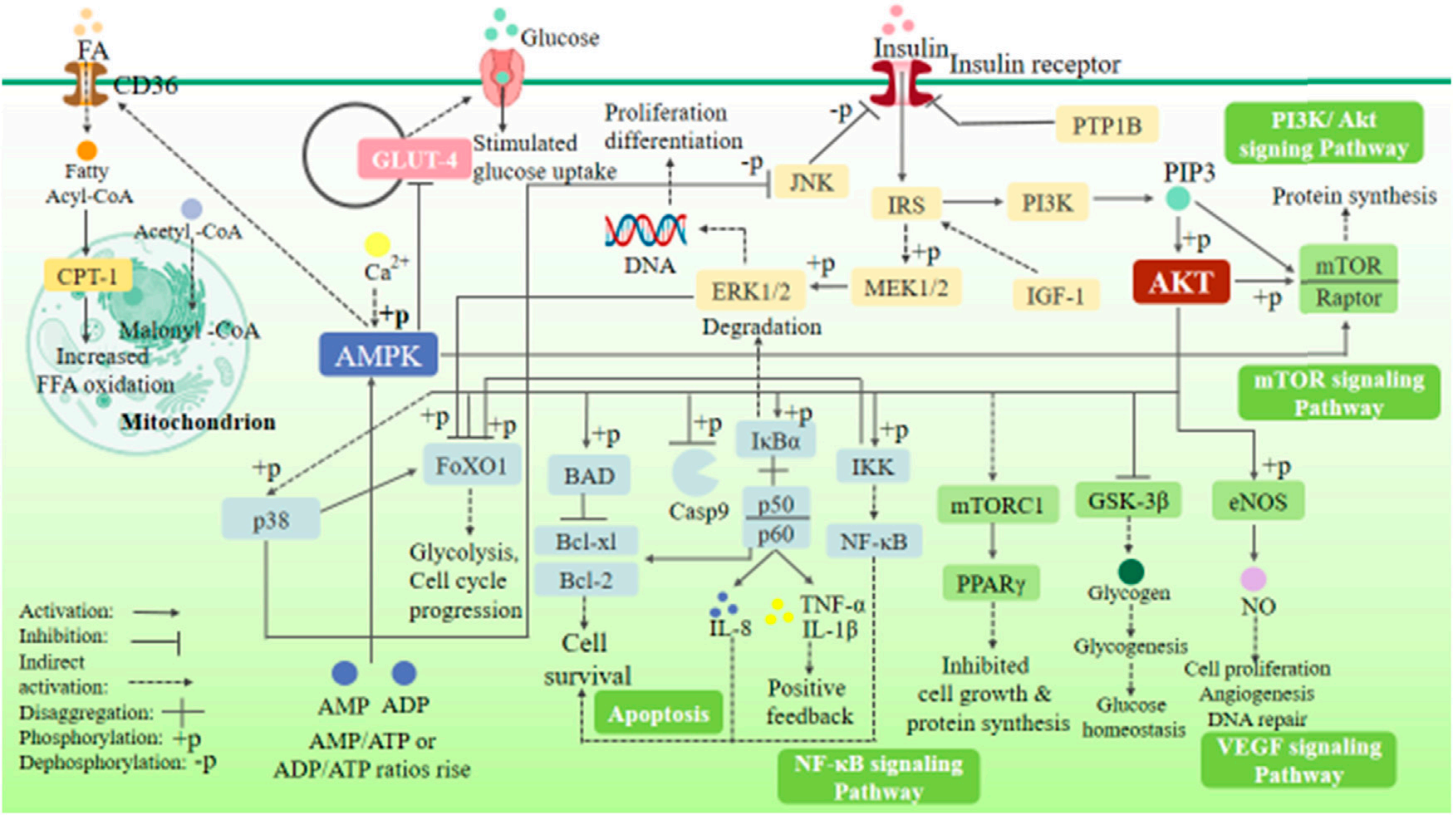
Signaling pathway cascade diagram.

After summarizing the action pathways, targets, and metabolic pathways of synthetic drugs, natural drugs, and a few derivatives mentioned above, it was found that most drugs regulate glucose metabolism, fatty acid oxidation, vascular endothelial function, and apoptosis mainly by acting on several signaling pathways or targets such as PI3K/Akt, AMPK, eNOS, NF-κB, and p38MAPK. Cascading the main signaling pathways (Figure 3), it is found that the activation of Akt is the core that affects other pathways and gene expression. Through the action of Akt on AMPK, MAPK, eNOS, NF-κB, and other pathways and targets, it can affect glycolysis, cell growth, protein synthesis, and other metabolic pathways. There is a direct or indirect relationship between each target and pathway, and they are inseparable, so we can consider the combination of drugs to exert the maximum efficacy and appropriately reduce the adverse reactions. For example, Pan et al. (Jing et al., 2019) used metformin combined with sitagliptin to treat T2DM patients with HF. After three-month treatment, they found that the combined treatment had significant therapeutic effect, which could improve cardiac function and effectively reduce blood glucose and the level of related inflammatory factors (Jing et al., 2019). Moreover, the prognosis was good, and the clinical effect was better than that of metformin alone. Feng et al. (Yanling, 2021) used atorvastatin combined with metformin to treat patients with diabetic cardiomyopathy, and the results showed that atorvastatin combined with metformin treatment could reduce the blood glucose level, improve heart function, and regulate blood lipids. In addition, many targets and pathways involved in Figure 3 can be used as new directions for the future development of T2DM complicated with HF drugs, such as the continued development of PPAR agonists and GLP-1 agonists.

(Figure 3 involves PI3K/Akt, AMPK, VEGF, NF-κB, MAPK, and mTOR signaling pathways. It can be seen that the core of insulin signaling pathways is the activation of Akt. By activating the PI3K/Akt signaling pathway, it acts on pathways and targets such as AMPK, MAPK, eNOS, and NF-κB and affects metabolic pathways such as glycolysis, cell growth, and protein synthesis to regulate heart function.)

## Conclusion and Prospects

It is certain that the insulin signaling pathway is a promising therapeutic direction for T2DM patients with HF. It can improve myocardial energy metabolism and IR and regulate cell apoptosis, inflammatory response, vascular endothelial function, and other ways to improve T2DM, thus protecting the heart and improving heart function. Different from the current combination therapy for T2DM complicated with HF, the regulation of insulin signaling pathway can provide targeted treatment for the special combination, improve the common physiological and pathological characteristics of the two diseases, delay the development of T2DM complicated with HF, and ensure a good prognosis. The synthetic drugs in this article are mostly listed drugs and have basically mature drug research. They can regulate blood sugar and blood lipids while treating HF. Compared with the previous drugs, natural drugs and their active ingredients not only can improve patient’s cardiac function and prognosis but also are safe and suitable for long-term treatment of HF patients.

Notably, it has certain defects for drug therapy based on the insulin signaling pathway. Regulating the insulin signaling pathway to treat T2DM complicated with HF mainly aims to improve myocardial energy metabolism disorder, so as to ensure adequate heart energy and recover heart function, which is a chronic treatment. In different types and stages of HF, myocardial energy metabolism presents different states. The treatment of HF by regulating the insulin signaling pathway should be adjusted according to the disease progress. Acute HF is a severe stress state with severe hyperglycemia (Hongji, 2018). Metabolic therapy based on insulin signaling is not applicable to T2DM complicated with acute HF, which is in critical condition and needs urgent treatment. This therapy is suitable for T2DM complicated with chronic HF or T2DM complicated with acute HF in the stable phase. Chronic HF can be divided into four stages, including A, B, C, and D. Drug therapy of insulin signaling pathway can be considered for A, B, C stages, especially in A and B stages. The reason is that stage A is mainly for the treatment of HF risk factors, and stage B is mainly to prevent and improve ventricular remodeling and prevent aggravation of heart failure symptoms (Osuna et al., 2018). In these two stages, the use of insulin signaling pathway drugs to treat the energy metabolism disorder and slow down the development of IR can effectively prevent the development of HF to the next stage. In the C and D stages, the disease is further aggravated, especially in the D stage. Most powerful drugs in conjunction with the application of mechanical assist devices, heart transplantation, ultrafiltration, and other treatment methods were used in these two stages, so that drug therapy based on the insulin signaling pathway is not suitable in two stages. Therefore, it is suitable to use insulin signaling pathway drug therapy in the early term and mid-term of T2DM complicated with HF, which can effectively control blood sugar and prevent the further development of HF.

The insulin signaling pathway has not yet been considered in the treatment of HF because it still has several problems that need to be clarified. First is the effect of excessive activation of insulin signaling pathway on HF. Studies have shown that over-expression of Akt can cause pathological myocardial hypertrophy (Dorn and Force, 2005). Whether the over-expression of other genes can harm the myocardium has not been clearly investigated. But as a potential risk factor, excessive activation of insulin signaling pathway may worsen heart failure. Second is the regulation of systemic insulin signaling pathway. While drugs regulate the myocardial insulin signaling pathway and improve myocardial IR, the systemic insulin signaling pathway is also affected. The disorder of systemic metabolic environment may trigger other diseases or affect the prognosis of HF. Third, although the drugs mentioned in this review can act through the insulin signaling pathway, most of them lack clinical trials in T2DM complicated with HF and cannot directly prove that they can treat HF through the insulin signaling pathway. In particular, clinical data on natural drugs are very scarce, with only a small amount in China. Moreover, due to the poor absorption and low bioavailability of natural drugs, further structural modifications are needed to obtain drugs more suitable for clinical use. Fourth, patients with T2DM complicated with HF need long-term medication and long-term treatment. Most of the drugs mentioned in this article have a short medication time in clinical trials, so the long-term effects and safety of these drugs need further research and demonstration. In the future, medical treatment of HF will be promoted along the direction of insulin signaling pathway. With more perfect and optimized research results and the advent of new drugs, the pattern of drug treatment dominated by the “Golden Triangle” in the past will gradually change, and the treatment of HF will usher in a more diversified landscape.
